# Role of Physical Exercise and Nutraceuticals in Modulating Molecular Pathways of Osteoarthritis

**DOI:** 10.3390/ijms22115722

**Published:** 2021-05-27

**Authors:** Alessandro de Sire, Nicola Marotta, Cinzia Marinaro, Claudio Curci, Marco Invernizzi, Antonio Ammendolia

**Affiliations:** 1 Department of Medical and Surgical Sciences, University of Catanzaro “Magna Graecia”, 88100 Catanzaro, Italy; nicola.marotta@unicz.it (N.M.); cinziamarinaro83@gmail.com (C.M.); ammendolia@unicz.it (A.A.); 2 Physical Medicine and Rehabilitation Unit, Department of Neurosciences, ASST Carlo Poma, 46100 Mantova, Italy; claudio.curci@asst-mantova.it; 3 Physical Medicine and Rehabilitation, Department of Health Sciences, University of Eastern Piedmont, 28100 Novara, Italy; marco.invernizzi@med.uniupo.it; 4 Translational Medicine, Dipartimento Attività Integrate Ricerca e Innovazione (DAIRI), Azienda Ospedaliera S.S. Antonio e Biagio e Cesare Arrigo, 15121 Alessandria, Italy

**Keywords:** exercise, physical activity, nutraceuticals, osteoarthritis, dietary supplements, inflammation, aging, inflammaging

## Abstract

Osteoarthritis (OA) is a painful and disabling disease that affects millions of patients. Its etiology is largely unknown, but it is most likely multifactorial. OA pathogenesis involves the catabolism of the cartilage extracellular matrix and is supported by inflammatory and oxidative signaling pathways and marked epigenetic changes. To delay OA progression, a wide range of exercise programs and naturally derived compounds have been suggested. This literature review aims to analyze the main signaling pathways and the evidence about the synergistic effects of these two interventions to counter OA. The converging nutrigenomic and physiogenomic intervention could slow down and reduce the complex pathological features of OA. This review provides a comprehensive picture of a possible signaling approach for targeting OA molecular pathways, initiation, and progression.

## 1. Introduction

Osteoarthritis (OA) is one of the most common degenerative musculoskeletal disorders, characterized by a progressive loss of joint cartilage, synovial inflammation, formation of osteophytes, and subchondral bone remodeling [1,2,3]. This detrimental condition has a complex pathogenesis due to its multifactorial nature [4,5,6]. More specifically, the aberrant expression of degradative proteases or catabolic mediators might be induced in the chondrocytes, which contribute to cartilage erosion [7,8]. This imbalance between anabolic and catabolic processes might damage the structural integrity of the joint cartilage, resulting in stiffness, pain, and limited range of motion (ROM) in the later stages of OA [9,10]. Thus, subsequent loss of function, increased disability, lower performance in the activities of daily living (ADL), and reduction of health-related quality of life (HRQoL) are common findings in these patients [11].

In this scenario, an early diagnosis supported by the detailed understanding of the molecular pathways underpinning OA could help to develop tailored conservative therapeutic approaches aimed to avoid surgical and joint replacement treatments [12]. To date, several non-surgical treatments have been proposed in the last years for OA patients, including pharmacological treatments (e.g., acetaminophen, nonsteroidal anti-inflammatory drugs (NSAIDs), duloxetine, and opioids) [13], intra-articular injections with hyaluronic acid and glucocorticoids [14], focal muscle vibration [15], intra-articular oxygen-ozone therapy [16], radiofrequency ablation of genicular nerves [17], adipose-derived mesenchymal stem cell therapy [18], and platelet-rich plasma injections [19]. However, physical exercise is recommended by several guidelines as the first-line intervention in OA patients [13,20,21], which plays a crucial role in the prevention and treatment of the disabling sequelae of this severe chronic disease [22,23].

Nowadays, other alternative therapeutic interventions to treat OA have come to the fore, such as nutraceuticals, defined as substances that can be considered as a food or part of a food and provides medical or health benefits, including OA prevention and treatment [24,25]. In vitro and in vivo studies showed that epigenetic changes are triggered by micronutrients commonly present in diets (e.g., vitamins, carotenoids, and flavonoids) that might modulate OA mediator pathways [26,27,28]. Indeed, myoblasts and chondrocytes seem to share similar pathological targets and pathways and close anatomical location, suggesting the possible existence of a paracrine network [29]. In this context, an adequate prescription of physical exercise and nutritional supplementation might have a positive impact not only in terms of OA molecular pathways modulation, but also in terms of functioning and HRQoL improvement.

Therefore, in the present comprehensive review, we sought to describe the state-of-the-art about the role that physical exercise and nutraceuticals might play in the complex management of OA, in terms of modulation of its molecular pathways.

## 2. Osteoarthritis Molecular Pathways

The pathogenic involvement in OA pathogenesis of pro-inflammatory cytokines released by chondrocytes and synoviocytes is well known and described in the scientific literature [7]. These cellular mediator patterns develop from cell signaling and gene expression pathways, which amplify the already altered cellular transduction, releasing additional inflammatory compounds and enzymes [30].

### 2.1. Reactive Oxygen Species

OA etiopathogenesis is influenced by several genetic and environmental factors not already fully explained. Recent studies have shown the involvement of oxidative stress and reactive oxygen species (ROS) in OA onset and progression [31]. ROS are unstable and highly reactive oxygen-containing free radicals combined with molecules to achieve chemical stability. Cells have developed antioxidant systems to scavenge ROS and maintain intracellular redox milieu. However, ROS are also key components of many physiological processes, and, at moderate concentrations, they act as indispensable second messengers. Their activity is based on the alterations of the cellular chemical environment, consisting of oxidative modification of proteins, influencing signal transduction, gene regulation, and cell cycling, in a complex process called redox biology [32]. The ability of cells to discriminate between the opposing effects of ROS is dependent on intensity, duration, and context of signaling and cellular redox status. When the cellular antioxidant capacity is insufficient to detoxify, ROS reacts with DNA, proteins, and lipids, disrupting their normal structure, impairing function, and leading to cytotoxicity in a process called oxidative stress [33].

Articular cartilage exists in relatively hypoxic conditions as a unique tissue thriving in a mechanically active environment. Despite living in a low O_2_ environment, chondrocytes are characterized by abundant and active mitochondria that contribute to adenosine triphosphate (ATP) production [34]. Disruption of mitochondrial function, leading to increased levels of intracellular ROS, has been hypothesized to disrupt cartilage homeostasis and is considered as one of the main contributors of OA-related cartilage damage [35].

In physiological conditions, ROS are produced at low concentrations in chondrocytes mitochondria through oxidative phosphorylation and in cytoplasm by nicotinamide adenine dinucleotide phosphate (NADPH) oxidase. The overproduction of ROS observed in OA impairs mitochondrial functions through mtDNA damage, inducing chondrocyte senescence and apoptosis, increasing cartilage degradation, and reducing matrix synthesis along with subchondral bone disfunction and synovial inflammation [36,37,38]. Evidence for ROS implication in cartilage degradation comes from the presence of lipid peroxidation products, nitrite, and nitrated products in the biological fluids in OA animal models [36]. On the contrary, antioxidant enzyme concentrations are decreased in OA patients, confirming the role that oxidative stress might play in OA pathogenesis [39].

Prolonged oxidative stress induces in the cartilaginous tissue the synthesis of large amounts of proteolytic enzymes, which in turn favors the shift towards catabolic reactions through several kinases such as p38 mitogen-activated protein kinase/nuclear factor kappa-light-chain-enhancer of activated B cells (p38MAPK/NF-κB) and activin receptor-like kinase 1 (ALK1) pathways [30]. In response to the proinflammatory stimuli, the overproduction of nitric oxide (NO) suppresses the cartilage matrix synthesis, enhances matrix metalloproteinases (MMPs) activity and induces chondrocyte apoptosis [40]. Taken together, all these mechanisms increase ROS concentrations leading to the membrane potential alterations that result in the release of cytochrome c and increase in Caspase-3 activity leading to apoptosis [41]. Lastly, oxidative stress-mediated inflammation is also responsible for the increased rate of hyaluronan degradation in synovial fluid [42].

On the other hand, chondrocytes respond to hypoxia and ROS damage, increasing the production of hypoxia-inducible factor-1 (HIF-1). HIF-1α can alleviate hypoxia-induced apoptosis, senescence, and matrix degradation in chondrocytes through mitophagy enhancing BCL2/adenovirus E1B 19-kDa-interacting protein 3 (BNIP3) expression [43].

### 2.2. Cellular Apoptosis

In the context of cellular autophagy, the phosphatidylinositol 3-kinase/protein kinase B (PI3K/AKT)/mechanistic target of rapamycin (mTOR) survival pathway plays a crucial role in OA pathogenesis. However, the role of Wnt/β-catenin and extracellular signal-regulated kinases (ERKs) pathway in OA pathogenesis is less elucidated [44]. In PI3K/mTOR signaling, AKT phosphorylated by PI3K modulates mTOR through a direct activation or an indirect block of one of its inhibitors. Activation of PI3K/mTOR pathway curbs the pro-apoptotic machinery resulting in enhanced cell survival through Caspase-3 inhibition [44,45]. Indeed, PI3K/mTOR is inactivated in IL-1b mediated chondrocyte apoptosis [46]. Alongside these mechanisms, Wnt proteins stand out through two characteristic pathways: β-catenin-independent pathway and β-catenin-dependent pathway [47]. In the independent process, the β-catenin undergoes phosphorylation, resulting in ubiquitination and consequent proteasomal degradation. On the other hand, in the β-catenin-dependent pathway, after the binding of Wnt ligands to the transmembrane frizzled (Fzd) receptor, the disheveled protein prevents the phosphorylation of β-catenin inducing the translocation into the nucleus where it stimulates the transcription of anti-apoptotic genes including c-Myc and cyclin D1 [48,49]. Lastly, recent studies seem to show that ERKs might be able to increase rather than decrease the levels of anabolic biomarkers in degenerative human chondrocyte [50,51,52].

### 2.3. Pro-Inflammatory Signaling

Alongside chondrocyte apoptosis, the scientific literature has recently focused on the mediation of inflammatory gene expression and inflammatory pathways role in OA pathogenesis. NF-κB is a protein complex that controls transcription of DNA and cytokine production [53]. In unstimulated cells, the NF-κB dimers are sequestered in the cytoplasm by a family of inhibitors called inhibitors of κB (IκBs) [54]. High Mobility Group Box 1 (HMGB1) is a non-histone DNA binding protein and is considered a damage-associated molecular pattern protein (DAMP) [55]. The binding of the extracellular ligand HMGB1 to the advanced glycation end product receptor (RAGE) has been described to activate IκB kinase (IKK), resulting in phosphorylation and degradation of IκBα [56].

Subsequently, p65 protein is released and phosphorylated for NF-κB heterodimer formation, and this complex is then translocated from the cytoplasm to the nucleus, inducing the expression of several genes such as cyclooxygenase (COX), Matrix metalloproteinases (MMPs), and pro inflammatory cytokines [10].

This pathway is associated with mitogen-activated protein kinase (MAPK) signaling which constitutes a family of serine/threonine kinases (p38 MAPK; c-Jun amino-terminal kinase (JNK); and ERK1/2) characterized by a multilevel crosstalk and activated by a large array of inflammatory and stressful conditions [57,58]. In addition to the ERKs, the activated p38 MAPK can translocate into the nucleus upregulating kinases and transcription factors promoting cell autophagy in some cells while enhancing survival, growth, and differentiation in other cells [57]. Lastly, the phosphorylation of JNK via specific tyrosine and threonine residues is able to increase pro-inflammatory gene expression [58].

### 2.4. Anti-Inflammatory Signaling

Nuclear erythroid factor 2 (Nrf2) is a distress mediator involved in oxidative stress reduction [59]. It operates as a transcription factor binding to the antioxidant response elements (AREs) in the promoter region of antioxidant genes. In unstimulated chondrocytes, Nrf2 is kept quiescent through the association with kelch-like ECH-associated protein 1 (Keap1) in the cytosol, hence restricting its translocation into the nucleus to bind the AREs [60]. DJ-1 is a sensitive redox protein that serves as a stabilizer of Nrf2; in DJ-1 knock-out animal models, the downregulation of Nrf2 could lead to an increase in oxidative stress. In the same context, heme oxygenase-1 (HO-1) is an Nrf2 downstream effector and both proteins are compensatory mediators that recognize pro-oxidative stressors and induce the production of antioxidant enzymes to neutralize the ROS [61]. Emerging evidence showed that the activation of DJ-1/Nrf2 signaling in chondrocytes is crucial to protecting these cells against oxidative insults, downregulating HMGB1 and NF-κB protein expression, and activating the PI3K/mTOR pathway [62].

In summary, the activation of NF kB, p38MAPK, and JNK is involved in the expression of several inflammatory genes that boost OA pathogenesis and progression. On the other hand, the upregulation of NRf2 could allow the expression of genes leading to a suppression of inflammation and inhibition of the activation of NF-κB. In this scenario, the PI3K/mTOR, Wnt, and ERK pathways appear to block cell apoptosis and promote proliferation.

## 3. Physical Exercise as a Modulator of Osteoarthritis Molecular Pathways

Physical exercise has been recognized as a safe, effective, and multifaceted therapeutic treatment to reduce pain and disability in OA patients [13,20,22,23,63].

The American College of Sports Medicine guidelines and EULAR recommend physical activity and exercise as positive and effective interventions on physical fitness as well as disease-specific and general outcomes in people with hip and knee OA. Moreover, these guidelines suggest that physical activity and exercise should be considered as integral parts of standard care [64,65].

Recommended exercise programs include land-based therapeutic exercise and water physiotherapy interventions [66,67]. Indeed, a recent systematic review confirmed that Pilates, aerobic, and strengthening exercise programs performed for 8–12 weeks, 3–5 sessions per week, with each session lasting 1 h, could be considered as an effective treatment in OA patients [68].

The exact protective mechanism of action of physical exercise on cartilage is not yet known, albeit several studies performed on animal models highlighted key molecules that could help define the best therapeutic exercise regimen.

Exercise modulates transcription in several metabolic pathways associated with extracellular matrix (ECM) biosynthesis and inflammation/immune responses in normal cartilage of rats undergoing treadmill walking (12 m/min, 45 min/day for 2, 5, or 15 days). More specifically, low-intensity exercises were able to regulate the different pathways involving PI3K-AKT, NF-κB, Ras, Rap, MAPK, cAMP, and Ptgs2. Moreover, exercise was able to suppress the expression of genes involved in ECM degradation, bone formation, and initiation of pro-inflammatory cascades, which are known to be upregulated in OA (Mmp9, Mmp8, Igf1, ColIa1, Adamts3, Adamts14). Conversely, exercise can upregulate genes involved in ECM synthesis that are commonly downregulated in OA (Chrdl2, Tnfrsf11b, Timp4, Thbs2, Tgfb1, Mmp3, Il1r1, Il1r2, Cilp, and Bmp5) [29]. Taken together, these results suggest a crucial and multifaceted role of physical exercise in several molecular pathways involved in OA pathogenesis and progression.

### 3.1. Chondroprotective Role of Physical Exercise

OA animal models have been widely used in the research field to investigate the role of physical exercise in modulating molecular pathways involved in OA pathogenesis, highlighting a positive effect on cartilage preservation.

Iijima and colleagues showed that low-speed treadmill walking exercise (12 m/min for 30 min/day, 5 days/week performed for 2–4 weeks) prevented the progression of post-traumatic bone and cartilage lesions and increased BMP-2 and BMP-6 expression in the chondrocytes of the joint superficial zone of 24 male Wistar rats with induced damage to knee joints [69].

Assis et al. studied the effects of aerobic exercise performed with treadmill, training 3 days/week at 16 m/min for 50 min/day for 8 weeks, on an animal experimental model of knee OA. At the end of the treatment protocol, the exercise group had a better pattern of cartilage organization with less fibrillation and irregularities along the articular surface and a lower degenerative process, compared with controls. Moreover, animals in the exercise group had a lower chondrocyte nuclear or nucleolar expression of IL-1β, Caspase-3, and MMP-13, confirming the ability of aerobic exercise to downregulate proinflammatory, proteolytic, and apoptotic pathways [70]. Similarly, treadmill exercise with moderate-intensity (18 m/min, 60 min/day, 5 days/week for 8 weeks) could have a chondroprotective effect in an OA animal model through the inhibition of NF-κB expression, resulting in a potent inhibition of MMP-13 gene expression [71].

Moderate-intensity exercise performed for only 4 weeks can also inhibit nuclear translocation of HDAC3/NF-κB complex, leading to the decreased expression of inflammatory proteins and cartilage protection in a rat model of OA. Similarly, in isolated primary rat chondrocytes mimicking OA-like pathologies, RGFP966, an HDAC3-specific inhibitor, could reduce IL-1β-induced inflammation and ROS production through the inhibition of nuclear translocation of NF-κB. Moreover, the same results were observed in a OA rat model, suggesting a role of RGFP966 in exerting exercise mediated chondroprotective effects [72]. Lastly, Castrogiovanni et al. demonstrated that a moderate-intensity physical activity protocol could lead to a decreased expression of OA-related biomarkers (IL-1, TNFα, MMP-13) and an increased expression of chondroprotective ones (IL-4, IL-10, and lubricin) in the synovium of an OA-induced rat model, highlighting the beneficial effects of exercise on cartilage preservation [73].

### 3.2. Anti-Inflammatory and Anti-Apoptotic Role of Physical Exercise on Murine Models

The anti-inflammatory effects of physical exercise in OA have been widely investigated on murine models. Here, a short review of the main findings showing the positive effects of physical exercise at different levels in OA damaged joints will be performed.

One hour treadmill activity showed an increase in Maresin-1 content, a strong anti-inflammatory molecule, in intra-articular lavage fluid of MIA-induced rat OA. Maresin-1 was able to decrease MMP13, activate the PI3k/Akt pathway, and suppress the NF-κB p65 pathway in IL-1β-induced rat fibroblast-like synoviocytes in vitro, suggesting a pivotal role of Maresin-1 in exerting the anti-inflammatory activity of exercise [74].

Similarly, four weeks of moderate exercise in a monosodium iodoacetate (MIA)-induced OA model determined IL-1β reduction and IL-4 increase in both serum and intra-articular lavage fluid, resulting in anti-inflammatory effect at cartilage tissue level. Furthermore, immunohistochemical assays showed that exercise markedly promoted the expression of autophagosome proteins LC3B, SQSTM1, and LC3II in the whole cartilage tissue, suggesting a positive role of exercise in autophagosome’s reaction to pathological stimuli [75].

Moderate intensity treadmill training in MIA-induced OA determined an increase in 15-hydroxyeicosatetraenoic acid (15-HETE) concentration in knee joint. 15-HETE is a well-known anti-inflammatory molecule and was also able to inhibit IL-1β-induced inflammation in primary chondrocytes and increase p-Akt levels in vitro. Moreover, 15-HETE injection in knee OA alleviated cartilage damage through the inhibition of MMP-13 expression and the concomitant increase in COL2 expression in joint cartilage tissue [76].

Other animal experimental models of OA included anterior cruciate ligament transection. A study performed on this specific animal model revealed that 4 months of moderate exercise determined the reduction of Caspase3 expression and concomitant overexpression of Hsp70, an antiapoptotic factor, in both superficial and deep zones of the knee medial compartment [77]. Similarly, in surgically-induced rat knee OA, low-speed treadmill exercise (12 m/min, 5 days/week for 4 weeks) increased pSmad-5, Id1 and BMP-2, BMP-4, BMP-6, and BMP receptor 2 expression in the superficial zone chondrocytes and suppressed cartilage degeneration [69].

In this scenario, it should be noted that physical exercise could provide beneficial effects in OA according to several factors, including adherence to exercise regimens, frequency of exercise, and actual loading of the symptomatic compartment [76,78]. Nam et al. [78] demonstrated that the extent of cartilage damage could play a key role in achieving the optimal effects of exercise. They conducted a study using a common treadmill exercise protocol (speed of 12 m/min for 45 min/day) after MIA-induction damage at knee level in rats, demonstrating that exercise significantly prevented the OA progression, albeit its efficacy appeared to be inversely related to the extent of cartilage damage. Transcriptome-wide gene expression analysis revealed that gentle treadmill activity started 1 day post–MIA OA induction significantly suppressed inflammation-associated genes through NF-κB network downregulation, with crucial effects on apoptosis and cell cycle (Cdkn1a and Bcl2), matrix breakdown (Mmp12 and Mmp14), and proinflammatory responses (IL15 and IL18) in murine models. On the other hand, a delayed intervention after grade 1 cartilage damage could be less effective in suppressing proinflammatory genes or upregulating matrix synthesis. These effects were mainly related to Sox9 suppression leading to the down-regulation of alkaline phosphatase, Cilp, Cilp2, and Mgp, which are crucial mediators required for correct matrix assembly [78].

### 3.3. Beneficial Effects of Physical Exercise on Osteoarthritis Patients

Evidence observed on rat and mouse OA models have provided further insights that have been translated to humans since the early disease stages. The beneficial effect of exercise has been already showed in subjects at high risk of developing OA and alterations in articular cartilage composition could be a good marker of early OA pathological modifications [79].

In a randomized trial, patients at a high risk of developing radiographic OA for partial medial meniscus resection were subjected to 4 months weight-bearing exercise protocol. Cartilage quality, comprising GAG, was evaluated through fixed-charge density tissue measurement by gadolinium-enhanced MRI (dGEMRIC). The exercise group showed an improvement in dGEMRIC, compared with controls (*p* = 0.036), suggesting that human cartilage has a potential to adapt to loading changes [80]. This adaptation consequent to exercises varies between different locations within the joint, and the largest beneficial effect is observed in the load-bearing cartilage, such as the lateral posterior cartilage [81].

dGEMRIC index changes were also investigated in the study of Munukka et al. The authors evaluated 87 postmenopausal women with mild knee OA performing leisure-time physical activity (LTPA). After a period of 12 months, a linear relationship between higher LTPA level and increased dGEMRIC index changes in the posterior region of the lateral and medial femoral cartilage, were observed. These results suggested that higher LTPA level is related to regional increases in estimated glycosaminoglycan content of tibiofemoral cartilage [82]. Similarly, a randomized controlled trial conducted on 43 post-menopausal women with mild knee OA performing lower limb aquatic resistance training, showed a significantly reduction of T2-MRI scores in the posterior region of the medial femoral condyle after 4 months of physical activity. A decrease in T2 scores is suggestive of improved integrity and orientation of the collagen fibers and a decrease in hydration of articular cartilage [82].

Another promising technique to assess OA-related cartilage modifications is the microdialysis method. Helmark et al. obtained information about the effect of a single-leg knee-extension protocol on cartilage biomarkers and cytokines, both inside the joint as well as in the synovium, in a group of women affected by knee OA. In this RCT, over a period of three hours, a single bout of mechanical loading resulted in a significant decrease in cartilage oligomeric matrix proteins (COMP) and an increase in the anti-inflammatory mediator IL-10 in both intra-articular and peri-synovium spaces, compared with controls (*p* < 0.05). Significant increases were also registered for IL-6 and IL-8 in both groups, whereas TNF-α increased peri-synovially in the experimental group only [83].

Similarly, a pilot RCT performed by Hunt et al. demonstrated that a 10-week exercise protocol, consisting of hip abductors, quadriceps, and hamstrings muscle strengthening, performed on seventeen subjects with radiographically confirmed medial tibiofemoral OA, could lead to a reduction in serum of the cartilage degradation marker COMP levels, compared with controls (2.11 log U/L vs. 2.36 log U/L; *p* < 0.04). Moreover, a significant relationship emerged between dynamic measure of knee joint load and the ratio between two molecular markers increased in OA patient biological fluids (the urinary C-telopeptide of type II collagen/serum C-propeptide of type II procollagen ratio β = 1.11, 95% CI = 0.15, 2.07; *p* = 0.04) [84]. Lastly, the combined nutritional and exercise intervention also seem to act on cartilage biomarkers. A recent study demonstrated that an 18-month period aerobic walking and strength training in combination with an intensive diet determined the reduction of serum collagen type I (C1M) and II (C2M) in obese elderly patients affected by tibiofemoral OA [85].

### 3.4. Physical Exercise as an Antioxidant Intervention

The role of physical activity on oxidative stress and redox biology balance has recently obtained an emerging interest in the scientific literature. It is already known that physical activity might improve antioxidant defenses and lowers lipid peroxidation levels in both adults and aged individuals [86]. At cellular level, the beneficial role of physical exercise in OA comes through the increased expression of chondrogenic transcription factors SOX9 in circulating mesenchymal progenitors associated with autophagy. Autophagy regulates mitochondrial activity in stem cells to provide the best metabolic conditions, thus limiting ROS production and preventing metabolic stress and genome damage [87].

Furthermore, aerobic exercise modulates the chondrocyte response according to the balance between redox milieu and mechano-transduction mechanisms. Chondrocytes subjected to mechanical stimulation activate the NF-κB signaling pathway, which is considered a possible link between loading and chondrocytic responses to proinflammatory cytokines [88].

Nrf2 is a stress response protein in OA chondrocytes with anti-oxidative and anti-apoptotic function and acts via ERK1/2/ELK1-P70S6K-P90RSK signaling axis in vitro [89]. Physical exercise could activate Nrf2 in response to the ROS increase, leading to the expression of antioxidant genes such as phase-II antioxidant enzymes such as glutathione and NADPH through Nrf2/Maf/ARE pathways activation [90].

Although researchers and clinicians have demonstrated a particular attention to the role of physical exercise antioxidant effects on OA, at present, evidence in literature about this issue is scarce. In MIA-induced OA rat model, 8 weeks of endurance exercise training (13 m/min, 50 min/day for 3 days/week) promoted free radicals generation that could stimulate both exercise adaptation and mitochondrial biogenesis. In this scenario, exercise promoted a significant increase in myeloperoxidase and superoxide dismutase activity in the articular capsule [91]. Similarly, a two-group cross-sectional study showed that strengthening exercises could changes antioxidant status of systemic markers in knee OA patients. More specifically, ten obese women affected by knee OA, following an acute bout of isokinetic exercise, showed an immediate post-exercise rise of non-enzymatic antioxidant capacity of serum sustained by scavenging activity of 1,1-diphenyl-2-picrylhydrazyl. This finding suggests that the up-regulation of the body’s antioxidant defense could be probably related to the mobilization of antioxidant reserves in plasma [92].

In the complex management of OA, physical exercise turned out to be effective also in synergy with other innovative therapies (i.e., mesenchymal cell implants or injections). In this scenario, autologous chondrocyte implants and bone marrow-derived mesenchymal cell implants or injections are innovative therapeutic interventions in OA focused on articular cartilage and subchondral bone damage treatment [93,94,95]. Studies in humans showed that exercise might enhance joint recruitment of bone marrow-derived mesenchymal stem cells and upregulates the expression of osteogenic and chondrogenic genes (Runx, MSx1, Sox9, COL2A1, ATG3), osteogenic microRNAs, and osteogenic growth factors (BMP2, BMP6) [87,96,97]. In experimental rodent models, physical exercise enhanced the osteogenic potential of bone marrow-derived mesenchymal stem cells and reduced their adipogenic potential. Moreover, exercise performed after stem cell implantation could enhance stem cell transplant viability [98,99,100,101,102]. Taken together, these results suggest a complex and multifaceted role of physical exercise on different antioxidant pathways corroborating the physiological basis supporting its efficacy in the treatment of OA.

### 3.5. Challenges and Potential Controversies

Despite its countless scientifically proven beneficial effects, physical exercise in certain conditions could be detrimental to articular cartilage health. Studies on human subjects reported that even moderate exercise, recommended as a treatment for OA, might increase markers of cartilage degradation in OA-affected joints [103,104]. Due to these controversies, the investigation of the imbalances in the molecular pathways involved in OA pathogenesis and progression could help in providing more insights about this controversial issue.

In an exercise-induced OA rat model, two weeks of 16 m/min treadmill activity showed that Ctnnb1, β-catenin, and Wnt-3a participate in the pro-inflammatory OA pathogenesis through an abnormal activation of the Wnt/β-catenin pathway due to excessive mechanical load [105]. Similarly a high-impact exercise protocol comprising flexion, extension, and compression of the limbs for two weeks resulted in IL-1β overexpression and IL-10 down-regulation in an advanced stage of OA induced in a rat model [106]. Siebelt et al. highlighted the impact of a 6-week treadmill intense exercise protocol in rats highlighting the loss of proteoglycan content in the exercised compared with sedentary OA joints induced by papain injection [104]. In surgically-induced rat knee OA, the same treadmill velocity was not able to modulate the effect of BMP pathway on OA pathological modifications, leading to the progression of morphological alterations [69]. Similarly, anterior cruciate ligament transected-animals running at a lower speed (18 m/min for 6 weeks) underwent advanced cartilage degradation, compared with the sedentary animals [107].

In humans affected by OA, Jayabalan et al. observed that 45 min of continuous walking resulted in a cumulative increase in COMP concentration, a marker of cartilage turnover, whereas interval walking was associated with COMP concentrations comparable to baseline. This study shed light about the possibility that incorporating resting periods in walking regimens may impact the potentially deleterious effects of longer continuous walking bouts on the knee joint [108].

In conclusion, the above-mentioned controversies might be due to the performance of high-intensity exercises for a long time. Indeed, they are not commonly recommended by the recent International Guidelines for frail OA patients [64,65].

## 4. Nutrigenomic: Role of Nutraceuticals on Osteoarthritis

Nutraceuticals are considered as dietary supplements including a concentrated form of a presumed bioactive substance, originally derived from a food, aimed to improve health status particularly in “active aging” [109]. In this scenario, nutraceuticals play a pivotal role on osteoarthritis pathogenesis with specific targets in modulating OA pathways (see Table 1).

Long-chain polyunsaturated fatty acids (PUFAs) include alpha-linolenic (ALA), eicosapentaenoic (EPA) and docosahexaenoic (DHA) and are structural components of cell membranes. However, they also exert their action in a high number of intracellular signaling and metabolic pathways [26]. PUFAs and their metabolites act as second messengers when intercalated in the cell membrane, inhibiting the expression of MMP-13 through the inactivation of the p38MAPK and NF-κB p65 pathway, and promoting the activation of the PI3k/Akt signaling [58,74].

In the recent literature, the isolated administration of Chondroitin Sulfate has been shown to increase the transcription of the gene for Cartilage Oligomeric Matrix Protein (COMP) and decrease the expression of Toll-like receptors, thus inhibiting the Nfkb1 pathway in chondrocytes [112].

Glucosamine (GlcN) is an amino monosaccharide component of glycosaminoglycan (GAG) chains, usually in the form of sulfate or hydrochloride salts derived from the chitin of the exoskeleton of crustaceans and from mushrooms [26]. Glucosamine promotes the proliferation of human osteoblasts modulating the mTOR pathway and enhancing β-catenin nuclear translocation with consequent activation of cell proliferation via cyclin D1 expression [49,121,137].

Sulforaphane is an isothiocyanate derived from its glucosinolate precursor glucoraphanin, which is found in edible cruciferous vegetables, especially in broccoli. It can ameliorate ROS-induced oxidative stress and cartilage matrix degradation suppressing pro-inflammatory cytokines such as IL-6, TNF-α, and IL-17, inhibiting the JNK signaling and activating Keap1/Nrf2 pathway [133,134,135].

Berberine is an isoquinoline alkaloid, produced by many plants, as Coptis japonica Makino, Coptis, Berberis petiolaris and B. vulgaris [138]. It modulates the PI3K/Akt and NF-κB pathways regulating cytoskeletal reorganization and dedifferentiation in chondrocytes, improving the progression of OA [111].

Apigenin is a natural product belonging to the flavone class that is the aglycone of several naturally occurring glycosides. As an isolated compound from Cirsium japonicum var. maackii, it blocks MMP3, MMP13, and COX-2 expression through Hif-2α inhibition via the NF-κB and JNK signaling pathways [110]. Therefore, downstream of the NF-κB and JNK signal expression might block the translation of the pro-inflammatory factor HIF2a.

Eupatilin is an O-methylated flavonoid present in Artemisia asiatica (Asteraceae), which suppresses the expression of pro-inflammatory genes in chondrocytes through the reduction of JNK phosphorylation, NF-κB, and MAPK activation pathway [28].

Other nutraceuticals that could have a role in modulating the OA pathways seem to be the green tea catechins (GTCs), which could protect against cartilage loss and reduce the progression of OA through the modulation of NF-κB inhibitor expression and targeting the PI3K/AKT pathway. Another main component of the green tea is the epigallo-catechin-3 gallate that might inhibit NF-κB nuclear translocation by blocking IkB degradation in human chondrocytes stimulated with IL-1β [122,123,124].

Genistein, an isoflavone from soy (Glycine max), inhibits the NF-κB pathway and the consequent expression of catabolic factors, while stimulating the expression of Ho-1, associated with the activation of Nrf-2 in human chondrocytes [118,119,120].

Furthermore, Jaceosidin, an extract of *Artemisia argyi*, showed to reduce the damage to OA cartilage by blocking the degradation of IκB and inhibiting the nuclear translocation of NF-κB [117].

Quercitin is a hydroxyflavonoid widely contained in the flowers, leaves, and fruits of different plants. It improved OA via dose-dependent effects on the Toll-like Receptor-4 (TRL-4)/NF-κB pathway in vivo, inducing a cytosolic down expression of TLR-4 [129].

Moreover, Wogonin is an O-methylated flavone present in *Scutellaria baicalensis* with a positive effect on OA, which may be related to the inhibition of pro-inflammatory genes expression via the NF-κB pathway [136].

Curcumin, the main polyphenol component from the roots of turmeric (*Curcuma longa*), improves the inflammatory pattern through NF-κB signaling inhibition and Nrf2/ARE signaling activation. In addition, curcumin inhibits cell autophagy via ERK1/2 signaling pathway activation [52,113,114,115,116].

Olive oil polyphenols (OOPs), as oleocanthal (OC), oleuropein (OP), tyrosol (TY), and hydroxytyrosol (HT), could modulate inflammatory and degenerative OA molecular patterns. OOPs inhibit pro-inflammatory genes expression through the suppression of NF-κB and MAPK activation, while enhancing Nrf-2 signaling [125,126,127,128].

Lastly, resveratrol, a phenol extracted from the skin of grapes, wines, mulberries, and peanuts, belongs to the phytoalexins that protect plants from microbial infections. Resveratrol increases cell proliferation via stimulation of Wnt signaling dependent pathway and might exert self-limiting mechanism of inflammation through PI3K/Akt and Nrf2 axis activation and concomitant NF-κB inhibition [47,86,139,140].

**Figure 1:**
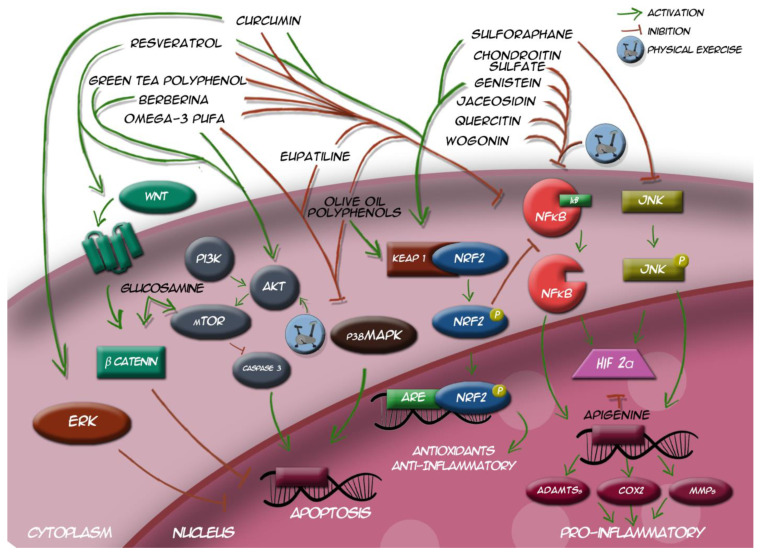
Involvement of nutraceuticals and exercise in molecular pathways for apoptotic, pro-, or anti-inflammatory signaling.

Therefore, nutraceuticals and physical exercise are involved in molecular pathways OA with apoptotic, pro-, or anti-inflammatory targets (see Figure 1 for further details).

## 5. Conclusions

In this comprehensive review, we described the main molecular pathways underpinning OA pathogenesis and progression, highlighting the different mechanisms of action and potential targets for innovative therapeutic interventions. Moreover, we described common targets of physical activity and nutraceuticals suggesting a possible therapeutical synergism of these two interventions. The decrease in ROS production upstream of physical exercise can guarantee a beneficial approach regardless of the nutraceutical chosen, whereas the mutual connections on a cellular mediator would suggest the composition of the compounds in an ad hoc logic.

In conclusion, despite the limitations related to the low characterizations of some molecular pathways, the current review provides intriguing insights regarding molecular pathophysiological mechanisms, innovative therapeutical target pathways, and synergistic interventions that could improve the complex framework of OA management.

## Figures and Tables

**Table 1:** Characteristics of the studies included with the nutrigenomic targets.

Nutraceutical	OA Pathways Involved	Modulation Action	Main Findings	Journal	Authors	Year
Apigenin	┤ HIF 2α	↓ ADAMTS-4 ↓ COX-2 ↓ MMP	Apigenin blocks osteoarthritis development as Hif-2α inhibitor.	*Journal of Cellular and Molecular Medicine*	Cho et al. [110]	2019
Berberine	→ PI3K/AKT ┤ NF-κB	↓ ADAMTS-4 ↓ COX-2 ↓ MMP	Berberine activates PI3K/Akt and NF-κB pathways.	*Phytomedicine*	Wong et al. [111]	2019
Chondroitin Sulfate	┤ NF-κB	↓ ADAMTS-4 ↓ COX-2 ↓ MMP	Separate administration of chondroitin sulfate raised expression of Comp and reduced TLRs, and NF-κB expressions in cartilage.	*Probiotics and Antimicrobial Proteins*	Korotkyi et al. [112]	2021
Curcumin	→ ERK1/2	↓ Chondrocyte Apoptosis	Curcumin inhibits apoptosis of chondrocytes through activation ERK1/2 signaling pathways induced autophagy.	*Nutrients*	Li et al. [52]	2017
	┤NF-κB	↓ ADAMTS-4 ↓ COX-2 ↓ MMP	Curcumin reduces inflammation in knee osteoarthritis through blocking TLR4/MyD88/NF-κB signal pathway.	*Drug Development Research*	Zhang et al. [113]	2018
	→ Nrf2/HO-1	↑ GPX1,3,4 ↑ SOD1 ↑ CAT ↑ GST	Curcumin inhibits chondrocytes inflammation through the Nrf2/ARE signaling pathway, thereby exerting cartilage protective effects.	*Cell Stress and Chaperones*	Jiang et al. [114]	2020
	┤ TLR4/NF-κB	↓ ADAMTS-4 ↓ COX-2 ↓ MMP	Curcumin improve neuroinflammatory process by reducing microglia/macrophage activation and neuronal apoptosis through a mechanism involving the TLR4/NF-κB signaling pathway in microglia/macrophages.	*International Journal of Molecular Sciences*	Panaro et al. [115]	2020
	┤ NF-κB	↓ ADAMTS-4 ↓ COX-2 ↓ MMP	Curcumin reduces expression of NF-κB and ROCK1.	*Journal of Cellular and Molecular Medicine*	Qiu et al. [116]	2020
Eupatilin	┤ NF-κB ┤ JNK ┤ p38MAPK	↓ ADAMTS-4 ↓ COX-2 ↓ MMP	Eupatilin suppressed expression of MMPs, ADAMTSs in chondrocytes by reducing JNK phosphorylation and NF-κB and MAPK signaling.	*Pharmaceuticals*	Lee et al. [117]	2021
Genistein	→ Nrf2/HO-1	↑ GPX1,3,4 ↑ SOD1 ↑ CAT ↑ GST	Genistein downregulates MMPs, ADAMTSs via NF-κB signaling pathway by blocking IκB degradation and activating Keap1/Nrf2 pathway.	*Nutrients*	Liu et al. [118]	2019
	┤ NF-κB	↓ ADAMTS-4 ↓ COX-2 ↓ MMP		*BioMed Research International*	Yaun et al. [119]	2019
				*Molecular Medicine Report*	Zou et al. [120]	2020
Glucosamine	→ mTOR	↓ Chondrocyte Apoptosis	Glucosamine promotes osteoblast proliferation by modulating autophagy via the mTOR pathway.	*Biomedicine & Pharmacotherapy*	Lv et al. [121]	2018
	→ Wnt/β-catenin	↓ Chondrocyte Apoptosis	GlcN increases β-catenin nuclear translocation, thus promoting chondrocyte proliferation.	*International Journal of Molecular Medicine*	Ma et al. [49]	2018
Green tea polyphenol	┤ NF-κB	↓ ADAMTS-4 ↓ COX-2 ↓ MMP	L-theanine inhibits upregulation of MMPs, as well as inhibiting NF-κB	*Nutrients*	Bai et al. [122]	2020
			Green tea catechins increase NF-κB inhibitors expression	*Antioxidants*	Luk et al. [123]	2020
	→PI3K/AKT	↓ FOX-O1	Epigallocatechin-3-gallate modulating AKT-FoxO1 via upregulating miR-486-5p.	*Archives of Biochemistry and Biophysics*	Chang et al. [124]	2020
Jaceosidin	┤ NF-κB	↓ ADAMTS-4 ↓ COX-2 ↓ MMP	Jaceosidin attenuates cartilage destruction by suppressing MMPs, ADAMTSs and the NFκB signaling pathway by blocking IκB degradation.	*Journal of Cellular and Molecular Medicine*	Lee et al. [117]	2019
Omega-3 PUFA	┤ p38MAPK	↓ Chondrocyte Apoptosis	PUFA inactivates of p38MAPK	*International Journal of Molecular Medicine*	Wang et al. [58]	2016
	→ PI3K/AKT ┤ NF-κB	↓ MMPs	PUFA metabolite suppresses MMP-13 secretion by activating PI3K/AKT pathway directly, while inhibiting NF-κB pathway.	*Connective Tissue Research*	Lu et al. [74]	2020
OOP	┤ NF-κB ┤ p38MAPK	↓ ADAMTS-4 ↓ COX-2 ↓ MMP	OOPs inhibited IL-1β-induced expression of inflammatory mediators through suppressing NF-κB and MAPK activation in chondrocytes.	*Food & Function*	Feng et al. [125]	2017
	→ Nrf2/HO-1 ┤ NF-κB	↓ ADAMTS-4 ↓ COX-2 ↓ MMP	OOPs can activate Nrf-2 signaling and the blockage of NF-κB nuclear translocation	*Cells*	Serrelli et al. [126]	2020
	┤ NF-κB	↓ ADAMTS-4 ↓ COX-2 ↓ MMP	OOPs reduce the inflammatory and catabolic factors mediated by NF-κB (IL-1ß, IL-6, COX-2 and MMP-3	*Aging*	Varela-Eirín et al. [127]	2020
	┤ NF-κB	↓ ADAMTS-4 ↓ COX-2 ↓ MMP	Mechanistically, OOPs exhibited an anti-inflammatory effect by inactivating the PI3K/AKT/NF-κB pathway.	*Journal of Cellular Physiology*	Chen et al. [128]	2021
Quercitin	┤ NF-κB	↓ ADAMTS-4 ↓ COX-2 ↓ MMP	Quercetin inhibits IL-1b and TNF-a production via TLR-4/NF-κB pathway.	*Journal of International Medical Research*	Zhang et al. [129]	2019
Resveratrol	→Wnt/β-catenin	↓ Chondrocyte Apoptosis	Rev increased osteoblastogenesis and bone formation through stimulation of Wnt signaling pathway.	*Journal of Cell Physiology*	Ashrafizadeh et al. [47]	2020
	→Nrf2/HO-1	↑ GPX1,3,4 ↑ SOD1 ↑ CAT ↑ GST	Res modulates the Nrf2 activation by inhibiting Keap1, Nrf2 gene expression, changing the upstream mediators of Nrf2, and potentiating the nuclear translocation of Nrf2.	*Biomedicine & Pharmacotherapy*	Farkhondeh et al. [130]	2020
	→PI3K/AKT	↓ FOX-O1	Resveratrol may exert anti-OA effect by enhancing the self-limiting mechanism of inflammation through TLR4/Akt/FoxO1 axis.	*Drug Design, Development and Therapy*	Xu et al. [131]	2020
	┤ NF-κB	↓ ADAMTS-4 ↓ COX-2 ↓ MMP	Resveratrol alleviates the interleukin-1β-induced chondrocytes injury through the NF-κB signaling pathway.	*Journal of Orthopaedic Surgery and Research*	Yi et al. [132]	2020
Sulforaphane	→ Nrf2/HO-1	↑ GPX1,3,4 ↑ SOD1 ↑ CAT ↑ GST	Sulforaphane ameliorates oxidative stress suppressing inflammatory cytokines and activating Keap1/Nrf2 pathway.	*Free Radical Biology and Medicine*	Yang et al. [133]	2020
			Sulforaphane inhibits the production of inflammatory cytokines.	*PLoS ONE*	Moon et al. [134]	2021
	┤ JNK	↓ ADAMTS-4 ↓ COX-2 ↓ MMP	Sulforaphane inhibits osteoclastogenesis by suppressing autophagy modulating JNK pathway.	*Molecules*	Lou et al. [135]	2021
Wogonin	┤ NF-κB	↓ ADAMTS-4 ↓ COX-2 ↓ MMP	Wogonin downregulates NF-κB pathway and genes involved in inflammatory-response.	*Nature: Scientific Report*	Khan et al. [136]	2017

Abbreviations: ADAMTS = A disintegrin and metalloproteinase with thrombospondin motifs; AKT = Tyrosine kinase A; CAT = Catalase; COX-2 = Cyclooxygenase-2; ERK = Extracellular signal-regulated kinases; FOX-O1 = Forkhead box protein O1; GPX = glutathione peroxidase; GST = Glutatione S-transferasi; HIF-2α = Hypoxia-inducible factor; HO-1 = Heme oxygenase 1; MAPK = Mitogen-activated protein kinases; MMP = Matrix metalloproteinase; mTOR = mammalian target of rapamycin; NF-κB = Nuclear factor kappa-light-chain-enhancer of activated B cells; Nrf2 = Nuclear factor-erythroid factor-2; OOP = Olive Oil Polyphenols; PI3K = Fosfoinositide 3-chinasi; PUFA = Polyunsaturated Fatty Acids; SOD = Superoxide dismutase; TLR4 = Toll-like receptor; Wnt = Wingless-related integration site.

## Data Availability

Not applicable.
